# Biomarker-based risk model to predict persistent multiple organ dysfunctions after congenital heart surgery: a prospective observational cohort study

**DOI:** 10.1186/s13054-023-04494-7

**Published:** 2023-05-20

**Authors:** Alexis L. Benscoter, Jeffrey A. Alten, Mihir R. Atreya, David S. Cooper, Jonathan W. Byrnes, David P. Nelson, Nicholas J. Ollberding, Hector R. Wong

**Affiliations:** 1 Division of Cardiology, Department of Pediatrics, Cincinnati Children’s Hospital Medical Center, University of Cincinnati College of Medicine, 3333 Burnet Ave, MLC 2003, Cincinnati, OH 45229 USA; 2 Division of Pediatric Critical Care Medicine, Department of Pediatrics, Cincinnati Children’s Hospital Medical Center, University of Cincinnati, Cincinnati, OH USA; 3 Division of Cardiology, Department of Pediatrics, University of Alabama at Birmingham, Birmingham, AL USA; 4 Division of Pediatric Critical Care Medicine, Department of Pediatrics, University of Kentucky, Lexington, KY USA; 5 Division of Biostatistics and Epidemiology, Department of Pediatrics, Cincinnati Children’s Hospital Medical Center, University of Cincinnati, Cincinnati, OH USA

**Keywords:** Cardiopulmonary bypass, Inflammation, Biomarkers, Risk stratification, Multiple organ dysfunction, Pediatric cardiac critical care

## Abstract

**Background:**

Multiple organ dysfunction syndrome (MODS) is an important cause of post-operative morbidity and mortality for children undergoing cardiac surgery requiring cardiopulmonary bypass (CPB). Dysregulated inflammation is widely regarded as a key contributor to bypass-related MODS pathobiology, with considerable overlap of pathways associated with septic shock. The pediatric sepsis biomarker risk model (PERSEVERE) is comprised of seven protein biomarkers of inflammation and reliably predicts baseline risk of mortality and organ dysfunction among critically ill children with septic shock. We aimed to determine if PERSEVERE biomarkers and clinical data could be combined to derive a new model to assess the risk of persistent CPB-related MODS in the early post-operative period.

**Methods:**

This study included 306 patients < 18 years old admitted to a pediatric cardiac ICU after surgery requiring cardiopulmonary bypass (CPB) for congenital heart disease. Persistent MODS, defined as dysfunction of two or more organ systems on postoperative day 5, was the primary outcome. PERSEVERE biomarkers were collected 4 and 12 h after CPB. Classification and regression tree methodology were used to derive a model to assess the risk of persistent MODS.

**Results:**

The optimal model containing interleukin-8 (IL-8), chemokine ligand 3 (CCL3), and age as predictor variables had an area under the receiver operating characteristic curve (AUROC) of 0.86 (0.81–0.91) for differentiating those with or without persistent MODS and a negative predictive value of 99% (95–100). Ten-fold cross-validation of the model yielded a corrected AUROC of 0.75 (0.68–0.84).

**Conclusions:**

We present a novel risk prediction model to assess the risk for development of multiple organ dysfunction after pediatric cardiac surgery requiring CPB. Pending prospective validation, our model may facilitate identification of a high-risk cohort to direct interventions and studies aimed at improving outcomes via mitigation of post-operative organ dysfunction.

**Supplementary Information:**

The online version contains supplementary material available at 10.1186/s13054-023-04494-7.

## Background

Cardiopulmonary bypass (CPB) potentiates a systemic inflammatory response in all patients, the degree of which varies based on many factors [1–9]. An exaggerated response, as seen in systemic inflammatory response syndrome (SIRS), can be detrimental and contributes to the development of multiple organ dysfunction (MODS), prolonged length of stay, and worse outcomes [5–7]. Almost all pediatric cardiac surgery patients meet criteria for organ dysfunction in the early postoperative period with ubiquitous inotropic and/or mechanical ventilator support, but children with optimal surgical interventions will begin to wean from postoperative support within the first few days. Failure to wean may represent persistent or progressive organ dysfunction, with risk of mortality increasing in conjunction with number of organ systems involved [10, 11]. Identifying patients at increased risk for persistent MODS due to an exaggerated inflammatory response to CPB could help guide clinical management, provide prognostic enrichment in future trials, and, ultimately, improve outcomes.

Sepsis and CPB both cause cellular injury and release of molecules that activate the innate and adaptive immune responses resulting in pro-inflammatory mediator upregulation [1, 3]. Research focusing on innate and adaptive immune gene expression and profiling in pediatric sepsis generated the Pediatric Sepsis Biomarker Risk Model (PERSEVERE) [12–20]. PERSEVERE and, more recently, PERSEVERE II have been utilized as risk-stratification tools to estimate probability of mortality and organ dysfunctions in pediatric septic patients [18]. Research on sepsis and CPB-mediated inflammation has identified significant overlap in inflammatory biomarker activation, including PERSEVERE biomarkers [5, 21–27]. We therefore posited that PERSEVERE biomarkers could be used to derive a unique risk model for early prediction of persistent MODS after CPB in pediatric patients.

## Methods

### Patients, samples, and data collection

The study was approved by the institutional review board at Cincinnati Children’s Hospital Medical Center. All patients under the age of 18 years old undergoing surgery requiring CPB for correction of congenital heart disease between November 2016 and November 2020 were screened for eligibility. Patients were only included for their index surgery to prevent re-enrollment of patients requiring reoperation for residual lesions while still recovering from their initial surgery. For patients with single ventricle physiology, each surgical stage was treated as a separate index surgery, i.e., stage 1 palliative surgery, Glenn operation, Fontan operation, and/or biventricular repair. Due to the short time frame between stage 1 and Glenn, Glenn candidates were screened prior to re-enrollment and were excluded if they met criteria for organ dysfunction at time of screening. Patients undergoing CPB for heart or lung transplantation, patients requiring immunosuppression, and patients with suspected or proven infection were excluded. Three-hundred and fifty-nine patient encounters (293 unique patients) were consented for the study. Of these, 306 encounters were included in the analysis, because both 4- and 12-h biomarker samples were collected within the specified time. Baseline demographic, clinical, and laboratory data needed to calculate severity of illness scoring and determine organ dysfunction were extracted from the electronic medical record (EMR). To minimize clinically unnecessary blood draws, laboratory data to assess for organ dysfunction were only collected at discretion of the managing clinical team.

### Definitions

The Society of Thoracic Surgery-European Association for Cardiothoracic Surgery (STAT) mortality category [28, 29] was used to account for risk related to surgical complexity. Pre- and postoperative severity of illness was assessed using Pediatric Risk of Mortality score III (PRISM III) [30]. Organ dysfunction was defined via adaption of Goldstein criteria [31] to account for differences in the postoperative congenital heart disease population when compared to the pediatric sepsis population, Additional file 1. Persistent MODS was defined a priori as dysfunction of 2 or more organ systems on postoperative day 5. As an additional measure of organ dysfunction, daily Pediatric Logistic Organ Dysfunction-2 (PELOD-2) scores were calculated preoperatively and for the first 5 postoperative days [32, 33].

### Clinical and surgical management

All patients received methylprednisolone (30 mg/kg) as part of the CPB circuit prime. Neonates and patients in the hospital prior to their scheduled operation received an additional dose of methylprednisolone (30 mg/kg) the morning of surgery (prior to CPB initiation). Choice of anesthesia was not standardized and left to the decision of the cardiac anesthesiologist. All patients received either modified ultrafiltration and/or continuous ultrafiltration intraoperatively, based on surgeon preference. The need for additional steroids and use of postoperative peritoneal dialysis was left to the discretion of the clinical team.

### Biomarker collection

Biomarkers were collected 4 and 12 h post-CPB, based on studies suggesting peak inflammation occurs within 24 h of CPB separation [4–6, 8, 21, 34]. Blood was collected within a ± 60 min window, spun down to serum, and stored at – 80 C until ready to be analyzed. Seven PERSEVERE biomarkers were measured in this study: granzyme B (GZMB), heat shock protein 70 kDa 1B (HSPA1B), interleukin 1α (IL-1α), interleukin 8 (IL-8), C-C chemokine ligand 3 (CCL3), C-C chemokine ligand 4 (CCL4), matrix metalloproteinase 8 (MMP-8). Serum biomarker concentrations were measured according to manufacturer’s instructions using the HSP2MAG-63K multiplex bead platform (MILLIPLEX™ MAP Human Sepsis Magnetic Bead Panel 2-Immune Response Multiplex Assay) designed by the EMD Millipore Corporation (Billerica, MA, USA).

### Statistical analysis

Descriptive statistical analyses were performed using R (version 4.0.4). Demographic, clinical, and biomarker data were described using medians with interquartile ranges (IQR), means with standard deviations, or frequencies with percentages as appropriate. Comparisons of data for patients with and without persistent MODS were performed using the Kruskal–Wallis, Chi-squared, or Fisher’s exact tests as appropriate. Multivariate regression analysis, controlling for clinical data, was performed to examine the relationship between biomarker concentrations at 4 and 12 h and risk of development of MODS.

Classification and regression tree (CART) analysis was used to determine biomarker cut-points and derive a decision tree (Salford Predictive Modeler v6.6, Salford Systems, San Diego, CA) [35]. Candidate prediction variables for derivation of the decision tree were as follows: all seven PERSEVERE biomarkers at 4 and 12 h time points, change in PERSEVERE biomarker levels from 4 to 12 h, age in months (included as both continuous and dichotomous variables), single ventricle status, history of prematurity, CPB time, maximum vasoactive inotropic score (VIS), and STS-EACTS mortality category. Clinical predictor variable selection was based on extant literature [36–39]. Tuning parameters determined a priori included: tenfold cross-validation, at least one of the paired terminal daughter nodes contains ≥ 5% of the subjects in the root node, and no predictor variables repeated within one of the two main branches. Performance of the decision tree was determined by generating a classification table of true versus predicted status and calculation of discrimination metrics including sensitivity, specificity, positive and negative predictive values, and area under the receiver operating curve (AUROC). We compared our prediction model, which we will refer to as PERSEVERE-CPB, to PRISM III and STS-EACTS mortality category, as they are widely accepted and validated risk assessment and severity of illness scoring systems of this patient population, using the AUROC, sensitivity, and specificity. We further compared PERSEVERE-CPB to the 24-h postoperative PELOD-2 score, as PELOD-2 is a validated scoring system for organ dysfunction [32].

Using risk categories (referred to as PERSEVERE-CPB risk category) stratified the cohort into risk category based on high-, intermediate-, and low-risk terminal nodes of our model. We then evaluated the association of risk category with administration of postoperative steroids for hypotension and clinical outcomes.

Finally, we performed an uncontrolled subanalysis comparing biomarker concentrations in subjects who received dialysis (peritoneal or continuous renal replacement therapy) within the first 24 h after surgery to assess the potential effect of dialysis on biomarker concentration.

## Results

**Fig. 1:**
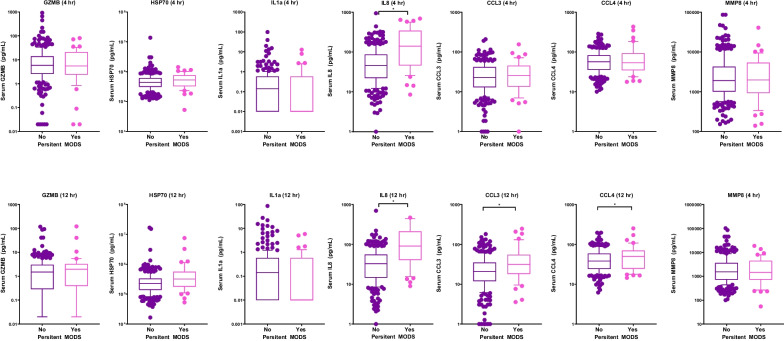
Comparison of biomarker concentrations in patients with and without persistent MODS. The serum interleukin-8 (IL-8) concentration was significantly elevated at 4 h after separation from cardiopulmonary bypass (CPB) in patients who developed persistent MODS and those who did not. IL-8, CCL-3, and CCL-4 concentrations at 12 h after separation from CPB were also significantly elevated in the cohort that developed persistent MODS compared to those that did not. Biomarker abbreviations displayed are as follows: GZMB, granzyme B; HSPA1B, heat shock protein 70 kDa 1B; IL-1α interleukin 1α; IL-8, interleukin 8; CCL3, C-C chemokine ligand 3; CCL4, C-C chemokine ligand 4; MMP-8, matrix metalloproteinase 8

Demographics, clinical characteristic, and biomarker concentrations of patients with and without persistent MODS are shown in Tables 1, 2 and Fig. 1, and Additional file 2. Of the 306 subjects with biomarkers drawn at both 4 and 12 h after separation from CPB, 43 (14.1%) had persistent MODS on POD 5. The cohort with persistent MODS was significantly younger, had a history of prematurity, had higher illness severity before and immediately after CPB, received more organ support, was more likely to receive steroids for post-operative hypotension, and had worse clinical outcomes. In multivariate logistic regression models, accounting for age less than 12 months, STAT mortality category, CPB time, and single ventricle status, IL-8 concentration at both 4 and 12 h was independently associated with risk of persistent MODS, as did 12-h concentrations of GZMB and CCL3, as shown in Table 2.

**Table 1:** Demographics and clinical characteristics

	All*	MODS*	No MODS*	*p* value
Number of subjects (%)	306	43 (14.0)	263 (86.0)	–
Age (months)	6 (3–42.9)	2 (0.2–5.3)	8 (3.9–48)	< 0.001
Number of females (%)	134 (43.8)	20 (46.5)	114 (43.3)	0.70
Race, number (%)				0.31
White, non-Hispanic	269 (87.9)	34 (79.1)	235 (89.3)	
White, Hispanic	6 (2.0)	2 (4.7)	4 (1.5)	
Black	23 (7.5)	6 (13.9)	17 (6.5)	
Other	8 (2.6)	1 (2.3)	7 (2.7)	
Number of neonates (%)	43 (14.1)	17 (39.5)	26 (9.9)	< 0.001
Number of single ventricle patients (%)	117 (38.2)	23 (53.5)	94 (35.7)	0.026
Number of infants (%)	182 (59.5)	38 (88.4)	144 (54.8)	< 0.001
Number of infants born premature (%)	45 (14.7)	13 (30.2)	32 (12.2)	0.002
STAT, number (%)				< 0.001
1	47 (15.4%)	3 (7.0)	44 (16.7)	
2	131 (42.8%)	12 (27.9)	119 (45.2)	
3	46 (15.2%)	3 (7.0)	43 (16.3)	
4	60 (19.6%)	15 (34.9)	45 (17.1)	
5	22 (7.2%)	10 (23.2)	12 (4.6)	
CPB time in minutes	138.0 (92.3; 183.0)	176.0 (112.0; 206.5)	132.0 (89.0; 179.0)	0.005
Number receiving MUF (%)	195 (63.7)	30 (69.8)	165 (62.7)	0.374
Pre-op PRISM III	2.0 (0.0; 3.0)	5.0 (3.0; 7.0)	0.0 (0.0; 3.0)	< 0.001
Post-op PRISM III	8.0 (6.0; 12.0)	13.0 (10.0; 16.0)	8.0 (5.0; 11.0)	< 0.001
PELOD-2 preoperative	0.0 (0.0; 2.0)	2.0 (0.0; 2.0)	0.0 (0.0; 2.0)	< 0.001
PELOD-2 24 h postoperative	4.0 (2.0; 6.0)	7.0 (5.0; 8.0)	4.0 (2.0; 5.0)	< 0.001
VIS at 4 h post-CPB	7.0 (5.0; 10.0)	8.0 (7.0; 11.8)	7.0 (4.5; 9.0)	0.007
Maximum VIS	7.0 (5.0; 15.4)	17.5 (14.5; 26.0)	7.0 (5.0; 12.5)	< 0.001
Lowest pH	7.29 (7.26–7.33)	7.25 (7.2; 7.3)	7.3 (7.3; 7.3)	< 0.001
Peak lactate	2.4 (1.6–4.0)	3.7 (2.3; 6.3)	2.2 (1.5; 3.8)	< 0.001
Number receiving PD or CRRT postoperative (%)	12 (3.9)	9 (20.9)	3 (1.1)	< 0.001
Number receiving steroids postoperative (%)	70 (22.9)	24 (55.8)	46 (17.5)	< 0.001
Number receiving steroids for hypotension postoperative (%)	27 (8.8)	13 (30.2)	14 (5.3)	< 0.001
Ventilator-free days	27.0 (26.0; 28.0)	17.0 (13.0; 23.0)	28.0 (26.0; 28.0)	< 0.001
Vasoactive-free days	26.0 (25.0; 27.0)	20.0 (14.5; 22.0)	27.0 (26.0; 27.0)	< 0.001
Number of in-hospital mortality (%)	7 (2.3)	6 (14.0)	1 (0.4)	< 0.001
Number alive and out of the hospital by POD 28 (%)	267 (87.3)	20 (46.5)	247 (93.9)	< 0.001
CICU LOS	3.0 (2.0; 8.0)	15.0 (11.0; 34.0)	3.0 (2.0; 4.5)	< 0.001
Hospital LOS	7.0 (4.0; 15.0)	24.0 (19.0; 67.0)	7.0 (4.0; 11.0)	< 0.001

All data are presented as median (interquartile range) unless specified; MODS: persistent multiple organ dysfunction at postoperative day 5; neonate: < 30 days old; infant: < 12 month old

*STAT* Society of Thoracic Surgery-European Association for Cardiothoracic Surgery mortality category, *CPB* Cardiopulmonary bypass, *PRISM III* Pediatric Risk of Mortality score III, *PELOD-2* Pediatric Logistic Organ Dysfunction Score-2, *VIS* Vasoactive inotropic score, *PD* Peritoneal dialysis, *CRRT* Continuous renal replacement therapy, *POD* Postoperative day, *CICU* Cardiac intensive care unit, *LOS* Length of stay

**Table 2:** Development of MODS based on PERSEVERE biomarkers

	OR (95% CI)	*p* value
Biomarkers at 4 h		
GZMB	0.79 (0.20; 1.18)	0.524
HSP70	0.94 (0.35; 1.35)	0.852
IL-1α	1.08 (0.50; 1.45)	0.684
IL-8	1.94 (1.41; 2.77)	< 0.001
CCL3	1.07 (0.74; 1.49)	0.700
CCL4	1.21 (0.88; 1.64)	0.210
MMP-8	1.15 (0.69; 1.60)	0.473
Biomarkers at 12 h		
GZMB	1.42 (1.04; 1.88)	0.012
HSP70	1.27 (0.89; 1.65)	0.081
IL-1α	0.69 (0.18; 1.23)	0.394
IL-8	11.42 (2.91; 57.11)	0.001
CCL3	1.36 (1.02; 1.84)	0.038
CCL4	1.27 (0.92; 1.71)	0.125
MMP-8	0.99 (0.46; 1.44)	0.963

Odds ratios (OR) obtained via logistic regression. Each biomarker was modeled separately. All models adjusted for age less than 12 months (infant), STAT mortality category, single ventricle status, and time (in minutes) on cardiopulmonary bypass

*CI* confidence interval, *GZMB* granzyme B, *HSPA1B* heat shock protein 70 kDa 1B, *IL-1α* interleukin 1α, *IL-8* interleukin 8, *CCL3* C-C chemokine ligand 3, *CCL4* C-C chemokine ligand 4, *MMP-8* matrix metalloproteinase 8

### Biomarker-based risk prediction model

**Fig. 2:**
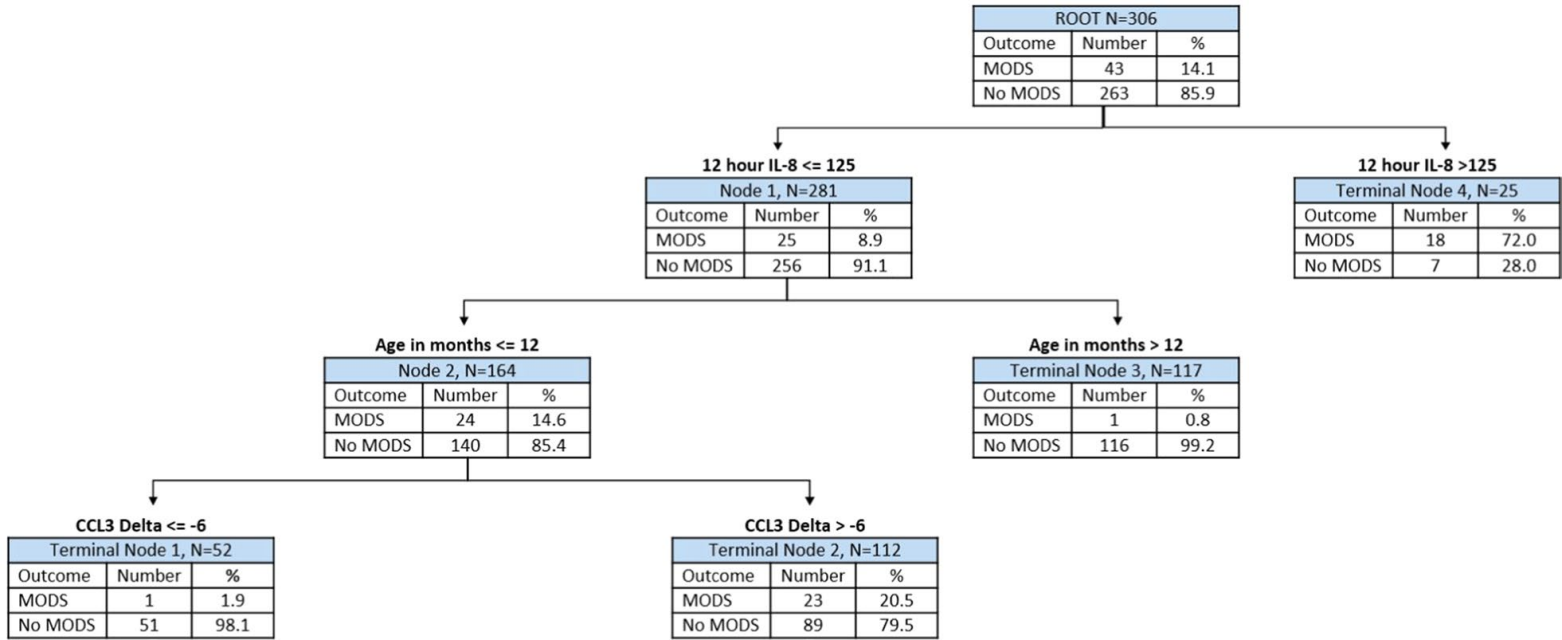
Derivation classification tree for PERSEVERE-CPB model. The classification tree consists of two biomarker-based decision rules and one clinically based decision rule. The 12-h interleukin-8 (IL8) serum concentration and the change in C-C chemokine ligand 3 (CCL3) serum concentration from 4 to 12 h were included. Each node contains the total number of subjects meeting the biomarker concentration or clinically based decision rule criteria, the number of subjects with or without persistent multiple organ dysfunction syndrome (MODS) at postoperative day (POD) 5, and the percentage of each respective outcome. Terminal nodes 1 and 3 were considered low-risk nodes, with subjects being less likely to develop persistent MODS. Terminal nodes 2 and 4 were considered high-risk and more predictive of development of persistent MODS. The area under the curve (AUC) for this tree was 0.86, with cross-validated estimate for AUROC of 0.75

Our newly derived PERSEVERE-CPB model is shown in Fig. 2. PERSEVERE-CPB included IL-8 concentration at 12 h, the change in serum concentration of CCL3 from 4 to 12 h, and infant age category (< 12 months). There were two low-risk terminal nodes (terminal nodes 1 and 3) in which subjects had < 2% risk of developing persistent MODS. There was one intermediate-risk node with 23 patients (20.5%) who developed persistent organ dysfunction (terminal node 2). There was one high-risk node with persistent organ dysfunction in 72% of patients (terminal node 4). PERSEVERE-CPB performed well at determining risk of persistent MODS with model characteristics shown in Table 3. IL-8 concentration at 12 h functioned as the upper tier decision rule, thus having the most predictive weight. Age less than 12 months was the second most important predictive variable, followed by change in the serum concentration of CCL3 from 4 to 12 h.

**Table 3:** Diagnostic test characteristics of PERSEVERE-CPB

Number of subjects	306
Number of true positives	41
Number of true negatives	167
Number of false positives	96
Number of false negatives	2
Sensitivity	95% (83; 99)
Specificity	64% (57; 69)
Positive predictive value	30% (23; 38)
Negative predictive value	99% (95; 100)
+ Likelihood ratio	2.6 (2.2; 3.1)
− Likelihood ratio	0.07 (0.02; 0.28)
AUC	0.86 (0.81;0.91)
Cross validation AUC	0.75 (0.68;0.84)

Numbers in parenthesis represent 95% confidence intervals

*AUC* Area under the curve, + *likelihood ratio* Positive likelihood ratio, − *likelihood ratio* Negative likelihood ratio

### Prediction performance

**Fig. 3:**
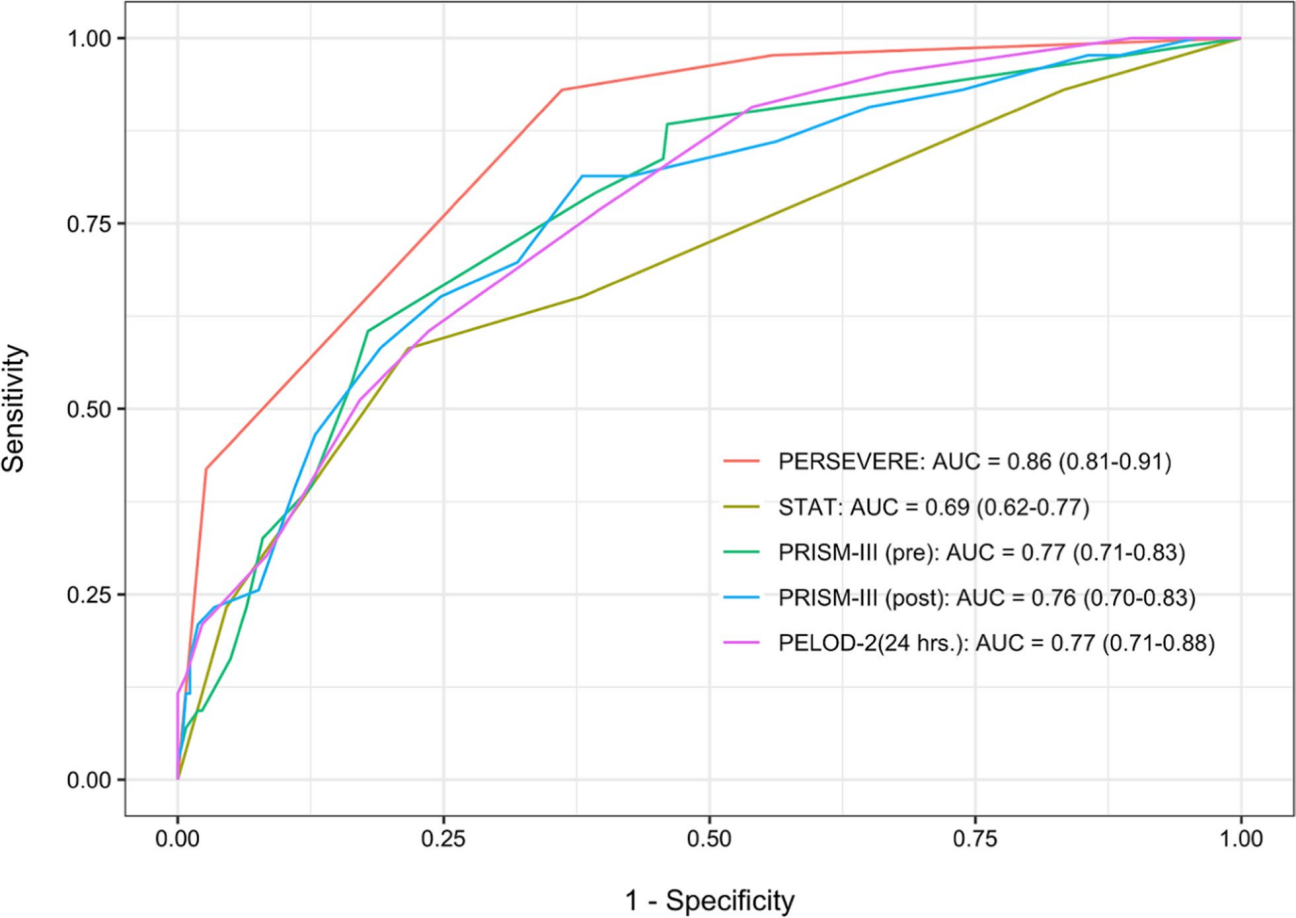
Comparison of PERSEVERE-CPB to validated risk-assessment tools to predict persistent MODS. PERSEVERE-CPB functioned well as a predictor of multiple organ dysfunction syndrome, with cross-validation area under the curve (AUC) that was comparable to validated risk-assessment tools in our cohort. PERSEVERE: PERSEVERE-CPB biomarker prediction model; STAT: Society of Thoracic Surgery-European Association for Cardiothoracic Surgery mortality category; PRISM-III (pre): Pediatric Risk of Mortality score calculated using preoperative data; PRISM-III (post): Pediatric Risk of Mortality score III calculated using data from the first 24 h after surgery; PELOD-2: Pediatric Logistic Organ Dysfunction Score-2

PERSEVERE-CPB had excellent performance for prediction of MODS: AUROC, 0.86 (95% CI 0.81; 0.91), Fig. 3. After cross-validation, our model’s corrected AUROC 0.75 (95% CI of 0.68–0.84) still had good performance. PERSEVERE-CPB performed favorably to other validated risk scoring systems for prediction of MODS in our study cohort: STAT, 0.69 (0.62; 0.77); preoperative PRISM III, 0.77 (0.71; 0.83), and postoperative PRISM III, 0.76 (0.70; 0.83). PELOD-2 calculated using data from the first 24 h after CPB had an AUROC of 0.77 (0.71; 0.88).

### Assessment of postoperative steroid need and outcome by PERSEVERE risk category

The portion of the cohort falling into the high-risk PERSEVERE-CPB category (terminal node 4 of model) were more likely to receive steroids for post-operative hypotension compared to those falling into the intermediate- and low-risk categories (35%, 22%, 2%, respectively; *p* < 0.001). The high-risk cohort also experienced longer duration of ventilator and vasoactive support, longer CICU and hospital stays, and had higher in-hospital mortality compared to those falling into intermediate- and low-risk categories, Table 4.

**Table 4:** Clinical outcomes by PERSEVERE-CPB Risk Strata

	High risk	Intermediate risk	Low risk	*p* value
Number receiving steroids postoperative (%)	14 (56.0%)	43 (25.6%)	13 (7.7%)	< 0.001
Number receiving steroids for hypotension postoperative (%)	7 (35.0%)	15 (22.4%)	5 (2.3%)	< 0.001
Ventilator-free days	21 (9, 25)	26 (24, 28)	28 (27, 28)	< 0.001
Vasoactive-free days	20 (8, 23)	25 (23, 27)	27 (26, 27)	< 0.001
Number of in-hospital mortality (%)	5 (20.0%)	2 (1.8%)	0 (0.0%)	< 0.001
Number alive and out of the hospital by POD 28 (%)	12 (48.0%)	91 (81.3%)	164 (97.1%)	< 0.001
CICU LOS	15 (11, 30)	5 (2, 11)	2 (1, 4)	< 0.001
Hospital LOS	25 (20, 57)	10 (6, 19)	6 (4, 9)	< 0.001

PERSEVERE-CPB risk category is based on terminal risk nodes from PERSEVERE-CPB model: Terminal node 4: high-risk; Terminal node 2: intermediate-risk; Terminal node 1 and 3: low-risk. All data is presented as median (interquartile range) unless specified; ventilator-free days: total days not receiving positive pressure ventilation out of 28 days; vasoactive-free days: total days not requiring vasoactive or inotropic medications out of 28 days

*POD* Postoperative day, *CICU* Cardiac intensive care unit, *LOS* Length of stay

### Biomarker concentrations in patients receiving dialysis

Dialysis, either continuous renal replacement therapy (CRRT) or PD, was used in 12 patients in the first 24 h after separation from CPB, with 9 being infants. Peritoneal dialysis catheters drained ascites without active dialysis in the remaining 34 neonates. Use of dialysis was associated with increased IL-8 at both 4 and 12 h post-CPB. CCL-3 concentrations were higher in the dialysis group, but only 12 h concentrations in the entire cohort were significant, Additional file 3.

## Discussion

Using inflammatory biomarkers and established clinical risk factors, we have derived a decision tree that is able to stratify patients by risk for developing persistent multiple organ dysfunction syndrome at post-operative day 5 after cardiopulmonary bypass surgery for congenital heart disease. Of the clinical risk factors and biomarkers included in this study, interleukin 8 (IL-8) concentration was the most important predictor of persistent MODS.

PERSEVERE-CPB allows a heterogenous cardiac surgery population to be stratified into high, intermediate, and low risk groups based on risk for persistent MODS. The model functions exceptionally well in identifying low risk patients, as illustrated by a high negative predictive value and low negative likelihood ratio. However, given the low positive predictive value and low positive likelihood ratio, PERSEVERE-CPB over-selects for MODS (approximately 31% false positive rate), which may limit its utility and result in overtreatment of the intermediate and high risk. Despite only a modest ability to predict MODS, our model enables the clinician to increase vigilance in a smaller cohort of patients, which has added importance as those falling into the high-risk PERSEVERE-CPB strata experienced worse clinical outcomes (longer duration of ventilator and vasoactive support, longer duration of stay, higher in-hospital morality) compared to the intermediate- and low-risk groups. This model has the potential to allow for early identification of patients categorized as low risk to receive standard of care supportive therapies, and those at intermediate or high risk to receive early targeted clinical interventions aimed at reducing the risk of MODS. Additionally, separation of low and higher risk cohorts may allow for prognostic enrichment in future clinical trials of interventions aimed at mitigating organ dysfunctions. However, without a rapid point of care PERSEVERE biomarker panel that allows for real time risk stratification, utility of PERSEVERE-CPB is limited. There are ongoing efforts at our institution focused on the development of a rapid point of care PERSEVERE biomarker panel. Once available, future studies will focus on timing and implementation of PERSEVERE-CPB in efforts to improve postoperative outcomes, including reduction in MODS. Future work with also focus on the addition of real-time physiologic and laboratory data to the model may improve the precision and specificity of this model.

For assessing risk of persistent MODS, PERSEVERE-CPB performed well when compared to existing pediatric critical care and cardiac surgery risk-assessment tools (STAT, PRISM III, PELOD 2). In particular, PERSEVERE-CPB performed similarly to the postoperative day one PELOD-2 score for predicting development of persistent MODS. Although STAT and PRISM III were primarily validated to predict risk of mortality and not MODS, the low mortality rate in our cohort did not allow us to develop a biomarker-based predictive model for in-hospital mortality.

IL-8 level functioned as the upper level decision rule, indicating that it played a key role in determination of risk for MODS. Almost 42% of patients who developed persistent MODS fell into terminal node 4, with an elevated 12 h IL-8 concentration. IL-8 is one of the more studied biomarkers of inflammation in patients after CPB. It is a neutrophil chemoattractant, plays a pivotal role in neutrophil activation, and is produced in large quantities by endothelial cells [40]. Elevated postoperative IL-8 has been associated with markers of low cardiac output (low mixed venous oxygen concentration and higher inotropic score) [41], development of postoperative acute kidney injury [26, 42, 43], increased duration of mechanical ventilation [22, 43, 44], and longer ICU length of stay [6]. The pathophysiologic role IL-8 plays in neutrophil/endothelium activation, bypass-mediated inflammation, and development of MODS warrants further examination, with obvious potential as a therapeutic target. In comparison, CCL3, or macrophage inflammatory protein 1α (MIP-1α), has not been extensively studied in bypass-mediated inflammation. During acute inflammation, CCL3 aids in the recruitment of leukocytes and plays a role in neutrophil infiltration [45, 46]. Since both PERSEVERE and PERSEVERE-II have demonstrated CCL3 plays a major role in discrimination of both mortality and multiple organ failure in severe pediatric sepsis [47], further investigation into the role of CCL3 in CPB-mediated inflammation and its contribution to development of organ dysfunction is warranted.

Age less than 12 months at time of surgery functioned as the second level decision rule in PERSEVERE-CPB. Younger age is known to be associated with increased morbidity after pediatric cardiac surgery [36, 37, 48], which is not a surprise given that infants and neonates undergo the most complex and highest risk surgeries. Future efforts to create risk models specific to infants and neonates could help determine if there are modifiable risk factors or potential therapeutic targets or if their increased risk is attributable to complexity of surgery and cardiac physiology (such as single ventricle physiology) alone.

Perioperative steroids are used in children undergoing CPB to blunt the bypass-mediated inflammatory response [49]. Non-uniform perioperative steroid administration in our population is a limitation of this study: while all patients received steroids, preoperatively hospitalized neonates and infants received steroids both before and during CPB, whereas all other patients received steroids only during CPB. It is unclear how additional steroid administration might have impacted these results, if at all. We acknowledge that perioperative steroids blunt the bypass-mediated inflammatory response; it is possible additional dosing may have resulted in even lower biomarker concentrations. Interestingly, the majority of the high-risk cohort (17 out of 20 subjects) were hospitalized neonates and infants which may suggest that inflammation has a bigger impact in outcome in this subset of patients, despite receiving two doses of steroids. Perhaps also supporting this theory, the high-risk cohort was more likely to receive steroids for hypotension in the first 24 h postoperative, which may reflect an enhanced inflammatory response leading to higher degree or longer lasting vasoplegia (Table 4).

Another limitation of the study was placement of peritoneal dialysis catheters in neonates, which is a standard practice at our institution. Use of peritoneal dialysis has been shown to decrease inflammatory cytokines after bypass and in other inflammatory states [50]. Unlike prior studies, use of dialysis was associated with increased IL-8 at both 4 and 12 h post-CPB in both the entire cohort and the neonatal subpopulation. CCL-3 concentrations were higher in the dialysis group, but only 12 h concentrations in the entire cohort were significant, Additional file 3. It is likely in this uncontrolled subanalysis that higher biomarker concentrations reflect treatment bias as opposed to a potentiating biomarker effect of peritoneal dialysis, as younger and higher risk patients/surgeries (with presumably more inflammatory CPB-response) are standardized to receive peritoneal catheters and undergo dialysis postoperatively. We cannot surmise the direct impact of peritoneal drainage and dialysis in this cohort without paired analysis and baseline biomarker levels. Future studies comparing postoperative inflammatory biomarker concentrations over time, use of dialysis, and correlation with risk of persistent MODS, particularly in the neonates and infants who, in this study, comprise a majority of the most at risk population, are warranted.

Also limiting our study, 3 subjects who developed MODS had a residual lesion or complication of care that contributed to prolonged need for mechanical ventilation and inotropic/vasopressor support. It would have been preferable to include only patients with MODS resulting from biologic and physiologic consequences of surgery and their intrinsic response to inflammation. Lastly, although the small number of events in this study prevented validation beyond a tenfold cross-validation procedure, we hope to be able to enhance this in a future, multicenter study. Cross-validation AUC for our model showed acceptable ability to predict persistent MODS, comparable to postoperative PRISM III and PELOD-2.

## Conclusions

Using known clinical risk factors and biomarkers of inflammation originally identified as key markers of inflammation in pediatric patients with septic shock, we have created a simple, biologically plausible model that accurately predicts risk of persistent organ dysfunction in pediatric patients after cardiac surgery for congenital heart disease. IL-8 concentration was the most predictive variable for development of MODS after CPB in our patient population; future efforts to better define CPB-related IL-8 pathophysiology and modifiable risk factors for IL-8 elevation after CPB are warranted.

## Supplementary Information

**Additional file 1:** Definitions of organ dysfunction. POD Post-operative day, SD Standard deviation, PaCO_2_ Arterial partial pressure of carbon dioxide, PaO_2_ Arterial partial pressure of oxygen, FiO_2_ Fraction of inspired oxygen, Cr Creatinine, ALT Alanine transaminase, NEC Necrotizing enterocolitis, INR International normalized ratio, GCS Glascow coma score.

**Additional** **file** **2:** Univariate association between PERSEVERE biomarkers and risk of Persistent MODS among children undergoing cardiopulmonary bypass. Odd ratiowith 95% confidence intervalsobtained via logistic regression. Each biomarker was modeled separately. ORs scaled to reflect one standard deviation increase in concentration. MODS Persistent multiple organ dysfunction at postoperative day 5, GZMB Granzyme B, HSPA1B Heat shock protein 70 kDa 1B, IL-1α Interleukin 1α, IL-8 Interleukin 8, CCL3 C-C chemokine ligand 3, CCL4 C-C chemokine ligand 4, MMP-8 Matrix metalloproteinase 8.

**Additional** **file** **3:** Interleukin-8 and chemokine ligand 3 concentrations in patients receiving dialysis within 24 h of surgery. All data presented as median. IL-8 Interleukin-8, CCL-3 C-C chemokine ligand 3.

## Data Availability

The datasets used and/or analyzed during this study are available from the corresponding author on reasonable request.

## Abbreviations

MODS: Multiple organ dysfunction syndrome
CPB: Cardiopulmonary bypass
SIRS: Systemic inflammatory response syndrome
PERSEVERE: Pediatric sepsis biomarker risk model
POD: Postoperative day
PaCO_2_: Arterial partial pressure of carbon dioxide
PaO_2_: Arterial partial pressure of oxygen
FiO_2_: Fraction of inspired oxygen
Cr: Creatinine
ALT: Alanine transaminase
NEC: Necrotizing enterocolitis
INR: International normalized ratio
GCS: Glascow coma score
CICU: Cardiac intensive care unit
STAT: Society of Thoracic Surgery-European Association for Cardiothoracic Surgery mortality category
PRISM III: Pediatric Risk of Mortality score III
PELOD-2: Pediatric Logistic Organ Dysfunction Score-2
VIS: Vasoactive inotropic score
LOS: Length of stay
GZMB: Granzyme B
HSPA1B: Heat shock protein 70 kDa 1B
IL-1α: Interleukin 1α
IL-8: Interleukin 8
CCL3: C-C chemokine ligand 3
CCL4: C-C chemokine ligand 4
MMP-8: Matrix metalloproteinase 8
PD: Peritoneal dialysis
CRRT: Continuous renal replacement therapy
