# Supplementary material for: Biomarker-based risk model to predict persistent multiple organ dysfunctions after congenital heart surgery: a prospective observational cohort study
Source: Crit Care. 2023 May 20;27:193. doi: 10.1186/s13054-023-04494-7 (PMC10199562; doi:10.1186/s13054-023-04494-7)
Supplement: Supplementary file 1 — Additional file 1: Definitions of organ dysfunction. POD Post-operative day, SD Standard deviation, PaCO2 Arterial partial pressure of carbon dioxide, PaO2 Arterial partial pressure of oxygen, FiO2 Fraction of inspired oxygen, Cr Creatinine, ALT Alanine transaminase, NEC Necrotizing enterocolitis, INR International normalized ratio, GCS Glascow coma score. [file 13054_2023_4494_MOESM1_ESM.docx]

**Additional File 1: Definitions of Organ Dysfunction**

| Organ System | Definition of Dysfunction |
| --- | --- |
| Cardiovascular | On vasoactive drugs by POD 5  or persistent lactatemia >5 mmol/L  or hypotension < 5^th^ percentile for age or systolic blood pressure < 2 SD below normal for age |
| Respiratory | Need for invasive or non-invasive ventilation by POD 5  or persistent respiratory acidosis with PaCO2 > 65 mmHg or 20 mmHg above baseline  or PaO2/FiO2 <300 torr in absence of cyanotic heart disease or preexisting lung disease |
| Renal | Cr >2 times upper limit of normal for age or 2-fold increase in baseline Cr, need for dialysis |
| GI/Hepatic | Total bilirubin > 4 mg/dL (outside of newborn period) or ALT 2 times upper normal limit for age, development of NEC |
| Hematologic | Platelet count < 80,000/mm^3^  or INR > 2 in a patient not on warfarin |
| Neurologic | GCS < 11 in a non-sedated patient  or acute mental status change with decrease in GCS of > 3 from baseline  or new cerebrovascular accident |

POD: post-operative day; SD: standard deviation; PaCO2: arterial partial pressure of carbon dioxide; PaO2: arterial partial pressure of oxygen; FiO2: fraction of inspired oxygen; Cr: creatinine; ALT: alanine transaminase; NEC: necrotizing enterocolitis; INR: international normalized ratio; GCS: Glascow coma score
