# Supplementary material for: Biomarker-based risk model to predict persistent multiple organ dysfunctions after congenital heart surgery: a prospective observational cohort study
Source: Crit Care. 2023 May 20;27:193. doi: 10.1186/s13054-023-04494-7 (PMC10199562; doi:10.1186/s13054-023-04494-7)
Supplement: Supplementary file 2 — Additional file 2: Univariate association between PERSEVERE biomarkers and risk of Persistent MODS among children undergoing cardiopulmonary bypass. Odd ratiowith 95% confidence intervalsobtained via logistic regression. Each biomarker was modeled separately. ORs scaled to reflect one standard deviation increase in concentration. MODS Persistent multiple organ dysfunction at postoperative day 5, GZMB Granzyme B, HSPA1B Heat shock protein 70 kDa 1B, IL-1α Interleukin 1α, IL-8 Interleukin 8, CCL3 C-C chemokine ligand 3, CCL4 C-C chemokine ligand 4, MMP-8 Matrix metalloproteinase 8. [file 13054_2023_4494_MOESM2_ESM.docx]

**Additional File 2: Univariate association between PERSEVERE biomarkers and risk of Persistent MODS among children undergoing cardiopulmonary bypass.**

|  | Biomarkers at 4 hours |  |
| --- | --- | --- |
|  | OR (95% CI) | p-value |
| GZMB | 0.81 (0.27;1.20) | 0.55 |
| HSP70 | 1.01 (0.61; 1.31) | 0.93 |
| IL-1α | 0.95 (0.44; 1.26) | 0.81 |
| IL-8 | 2.42 (1.78; 3.42) | <0.001 |
| CCL3 | 1.13 (0.82; 1.49) | 0.42 |
| CCL4 | 1.28 (0.96; 1.68) | 0.07 |
| MMP-8 | 0.91 (0.55; 1.25) | 0.66 |
|  | **Biomarkers at 12 hours** |  |
|  | **OR (95% CI)** | **p-value** |
| GZMB | 1.14 (0.85; 1.45) | 0.30 |
| HSP70 | 1.12 (0.82; 1.42) | 0.37 |
| IL-1α | 0.68 (0.16; 1.18) | 0.46 |
| IL-8 | 32.97 (8.73; 154.67) | <0.001 |
| CCL3 | 1.60 (1.23; 2.11) | 0.001 |
| CCL4 | 1.31 (0.98; 1.73) | 0.05 |
| MMP-8 | 0.83 (0.41; 1.20) | 0.48 |

Odd ratio (OR) with 95% confidence intervals (95% CI) obtained via logistic regression. Each biomarker was modeled separately. ORs scaled to reflect one standard deviation increase in concentration (pg/mL). MODS: persistent multiple organ dysfunction at postoperative day 5; GZMB, granzyme B; HSPA1B, heat shock protein 70 kDa 1B; IL-1α interleukin 1α; IL-8, interleukin 8; CCL3, C-C chemokine ligand 3; CCL4, C-C chemokine ligand 4; MMP-8, matrix metalloproteinase 8
