# Supplementary material for: Biomarker-based risk model to predict persistent multiple organ dysfunctions after congenital heart surgery: a prospective observational cohort study
Source: Crit Care. 2023 May 20;27:193. doi: 10.1186/s13054-023-04494-7 (PMC10199562; doi:10.1186/s13054-023-04494-7)
Supplement: Supplementary file 3 — Additional file 3: Interleukin-8 and chemokine ligand 3 concentrations in patients receiving dialysis within 24 h of surgery. All data presented as median. IL-8 Interleukin-8, CCL-3 C-C chemokine ligand 3. [file 13054_2023_4494_MOESM3_ESM.docx]

**Additional File 3: Interleukin-8 and chemokine ligand 3 concentrations in patients receiving dialysis within 24 hours of surgery**

| **Entire Cohort (n=306)** | | | |
| --- | --- | --- | --- |
|  | **Dialysis** | **No dialysis** | **p-value** |
| **IL-8 concentration (pg/mL)**  **4 hours**  **12 hours** | 245.7 (105.3;354.2)  140.1 (73.1;313.4) | 49.4 (23.9;94.3)  35.4 (15.9;61.9) | <0.001  <0.001 |
| **CCL-3 concentration (pg/mL)**  **4 hours**  **12hours** | 37.8 (29.6;58.3)  61.4 (43.7;76.8) | 22.9 (13.1;41.8)  22.5 (12.9;35.1) | 0.06  0.001 |
| **Neonates only (n=43)** | | | |
|  | **Dialysis** | **No dialysis** | **p-value** |
| **IL-8 concentration (pg/mL)**  **4 hours**  **12 hours** | 343.8 (185.6;374.9)  179.1 (102.3;341.9) | 98.0 (56.3;168.1)  71.9 (41.2;99.2) | 0.004  0.002 |
| **CCL-3 concentration (pg/mL)**  **4 hours**  **12hours** | 37.1 (31.0;65.8)  67.8 (46.9;76.5) | 32.7 (14.5;58.4)  31.1 (22.5;66.5) | 0.366  0.101 |

All data presented as median (interquartile range). IL-8: interleukin-8; CCL-3: C-C chemokine ligand 3
